# Physicochemical and Volatile Compounds Analysis of Fruit Wines Fermented with *Saccharomyces cerevisiae*: FTIR and Microscopy Study with Focus on Anti-Inflammatory Potential

**DOI:** 10.3390/ijms25115627

**Published:** 2024-05-22

**Authors:** Paweł Paśko, Agnieszka Galanty, Tomasz Dymerski, Young-Mo Kim, Yong-Seo Park, Patricia Cabrales-Arellano, Victor Velazquez Martinez, Efren Delgado, Mikołaj Gralak, Joseph Deutsch, Dinorah Barasch, Alina Nemirovski, Shela Gorinstein

**Affiliations:** 1 Department of Food Chemistry and Nutrition, Faculty of Pharmacy, Jagiellonian University Medical College, 30-688 Kraków, Poland; p.pasko@uj.edu.pl; 2 Department of Pharmacognosy, Faculty of Pharmacy, Jagiellonian University Medical College, 30-688 Kraków, Poland; agnieszka.galanty@uj.edu.pl; 3 Department of Analytical Chemistry, Faculty of Chemistry, Gdańsk University of Technology, 80-233 Gdańsk, Poland; tomasz.dymerski@pg.edu.pl; 4 Department of Eat Out Culinary and Start Up, Mokpo Science University, Mokpo 58758, Republic of Korea; bliss0816@hanmail.net; 5 Department of Horticultural Science, Mokpo National University, Muan, Jeonnam 58554, Republic of Korea; ypark@mokpo.ac.kr; 6 Biology Department, Eastern New Mexico University, Portales, NM 88130, USA; cristian.cabralesarellano@enmu.edu; 7 Food Science and Technology, Department of Family and Consumer Sciences, New Mexico State University, Las Cruces, NM 88003, USA; yambe@nmsu.edu (V.V.M.); edelgad@nmsu.edu (E.D.); 8 Center of Excellence in Sustainable Food and Agricultural Systems, New Mexico State University, Las Cruces, NM 88003, USA; 9 Department of Physiological Sciences, Warsaw University of Life Sciences—SGGW, 02-787 Warsaw, Poland; mikolaj_gralak@sggw.edu.pl; 10 Institute for Drug Research, School of Pharmacy, Faculty of Medicine, The Hebrew University of Jerusalem, Jerusalem 9112001, Israel; dinorah.barasch@mail.huji.ac.il (D.B.); alina.nemirovskai@mail.huji.ac.il (A.N.)

**Keywords:** kiwifruit, pomegranate, persimmon, fruit wines, anti-inflammation, polyphenols, FTIR, SEM, volatile compounds

## Abstract

The growing trend in fruit wine production reflects consumers’ interest in novel, diverse drinking experiences and the increasing demand for healthier beverage options. Fruit wines made from kiwi, pomegranates, and persimmons fermented using *S. bayanus* Lalvin strain EC1118 demonstrate the versatility of winemaking techniques. Kiwifruit, persimmon, and pomegranate wines were analyzed using HPLC and GC-TOFMS analyses to determine their concentrations of phenolic acids and volatile compounds. These results were supported by Fourier transform infrared (FTIR) spectroscopy to characterize and compare chemical shifts in the polyphenol regions of these wines. The wines’ characterization included an anti-inflammatory assay based on NO, TNF-alpha, and IL-6 production in the RAW 264.7 macrophage model. FTIR spectroscopy predicted the antioxidant and phenolic contents in the wines. In terms of polyphenols, predominantly represented by chlorogenic, caffeic, and gallic acids, pomegranate and kiwifruit wines showed greater benefits. However, kiwifruit wines exhibited a highly diverse profile of volatile compounds. Further analysis is necessary, particularly regarding the use of other microorganisms in the fermentation process and non-*Saccharomyces* strains methods. These wines exhibit high biological antioxidant potential and health properties, providing valuable insights for future endeavors focused on designing healthy functional food products.

## 1. Introduction

Standard grape wines, when consumed in moderation, may demonstrate potential health benefits, due to their high polyphenolic content and antioxidant properties [1]. This may contribute to the reduced risk of some chronic diseases, including cardiovascular disorders or type 2 diabetes [1]. Although red and white wines are still mostly prepared from grapes, in recent years there has been a growing interest in producing unique wines from other fruits, such as apples, pears, apricots, peaches, cherries, and varied berries [1,2]. Many tropical and subtropical fruits, including oranges, mangoes, bananas, and pineapples, started to be introduced to the wine market as an attractive addition to the human diet, with a huge amount of valuable beneficial compounds rarely found in typical grape wines, but also rich in volatile compounds which cause a significant impact on their aroma and unique colors [2,3,4]. These alternative fruit wines offer a distinct flavor profile and often boast higher levels of vitamins, minerals, and antioxidants compared to traditional grape wines.

Exotic fruits, starting from citruses such as red grapefruits, positively influence serum triglyceride levels and serum antioxidant activity in patients suffering from coronary atherosclerosis. Additionally, as shown in recent studies, exotic fruits such as persimmon, kiwifruit, and pomegranate are great sources of bioactive substances [4,5]. The impact of different cultivars of kiwifruit on the bioavailability of minerals in rats who were fed atherogenic diets enriched with these fruits was proved, which may be related to the presence of polyphenols and tannins [2]. What is important is the bioactivity of the investigated fruits after wine preparation [3]. Rodríguez et al. [6] highlighted that the fermentation of fruits may have a significant impact on the synthesis of different aroma compounds or their precursors (organic acids, acid, esters, ketones, alcohols, and terpenes), related to the flavors and odors of the final product. The fermentation process may also increase antioxidant activity as an effect of producing or releasing bioactive compounds such as peptides, vitamins, amino acids, and phenolics.

Exotic fruit wine production provides a practical solution for utilizing unmarketable fruits by converting them into nutrient-rich beverages containing polypeptides, amino acids, and organic acids that are readily absorbed by the human intestine. The use of commercial yeast strains, while ensuring wine quality, also facilitates home production of these wines, making it easier to achieve desired aroma and flavor profiles. Exotic fruit wines, renowned for their interesting phytochemical content, typically rely on *Saccharomyces cerevisiae* during fermentation. This widespread practice in the fruit wine industry helps suppress wild microflora and ensures consistent fermentation outcomes [7,8,9,10]. *S*. *cerevisiae*, commonly used in wine fermentation, also enhances the bioaccessibility of bioactive molecules such as flavonoids, anthocyanins, and resveratrol through biosorption. This process allows the yeast to function as a delivery system for these compounds, increasing their functionality in food products and improving bioaccessibility post-digestion. The effectiveness of phenolic compounds, known for their bioactive properties, heavily depends on their bioavailability—how well they are extracted and absorbed from the food matrix during digestion, which influences their health benefits [11]. The integration of the biosorbed phenolic compounds in *S. cerevisiae* suggests promising applications in food enhancement, utilizing this yeast strain not only for fermentation but also as a vehicle for delivering health-promoting compounds effectively [12].

The trend towards producing fruit wines reflects consumers’ interest in novel and diverse drinking experiences, as well as the increasing demand for healthier beverage options. Fruit wines made from kiwi, pomegranates, and persimmons display the versatility of winemaking techniques and highlight the rich biodiversity of fruits available for vinification. As consumers become more adventurous in their wine choices, wineries are innovating by experimenting with non-traditional fruit varieties to create unique and memorable wine offerings. Overall, the production of fruit wines involves a delicate balance of using traditional techniques and exploring new scientific approaches to maximize flavor while maintaining the nutritional integrity of the wine.

Our study focused on three exotic fruit wines produced from kiwifruit, persimmon, and pomegranate, lately implemented to the market, and the comparison of their phenolic acid and volatile compound content, and anti-inflammatory properties in vitro. Moreover, the physicochemical and textural properties, and the ultrastructure of the wines were investigated, using a method based on the combination of Fourier transform infrared (FTIR) spectroscopy, confocal microscopy, and scanning electron microscopy (SEM) approaches, respectively.

## 2. Results and Discussion

### 2.1. Polyphenolic Compounds Analysis by HPLC Supported by FTIR and Confocal Microscopy

#### 2.1.1. HPLC Analysis

In all evaluated fruit wines, four phenolic acids were detected (chlorogenic, gallic, caffeic, and protocatechuic acid). Additionally, *p*-coumaric acid was found only in pomegranate wine. The concentrations of phenolic acids identified in wines are provided in Table 1.

Regarding pomegranate wine, gallic acid was the dominant phenolic compound, followed by chlorogenic acid. Akalın et al. [13] also found gallic acid to be the most abundant compound, followed by hydroxycinnamic acid. Additionally, caffeic, *p*-coumaric, and vanillic acid were noted in this wine. Poyrazoğlu et al. [14] determined the quantities of different phenolic compounds in juices and identified gallic acid as the greatest phenolic compound. In pomegranate wine, punicalagin, ellagic acid, coumaric acid, and gallic acid were noted as the most abundant phenolic compounds [13]. Generally, persimmon wine contained the lowest sum of phenolic acids (0.65 mg/g), with gallic acid as the most dominant, followed in descending order by chlorogenic acid, caffeic, and finally, protocatechuic acids. Liu et al. [15] obtained comparable results from a qualitative and quantitative profile of phenolic acids in persimmon wine. In kiwi wine, the amount of phenolic acids was four times lower as compared to pomegranate. The most abundant compound was gallic acid followed by protocatechuic and chlorogenic acids. Zhou et al. [16] determined phenolic compounds in kiwifruit wines and noted gallic acid, protocatechuic acid, catechins, chlorogenic acid, epicatechin, coumaric acid, and ferulic acid. For Hongyang kiwi wine, the most abundant phenolic compounds were catechins and chlorogenic acid.

#### 2.1.2. FTIR Analysis

**Figure 1:**
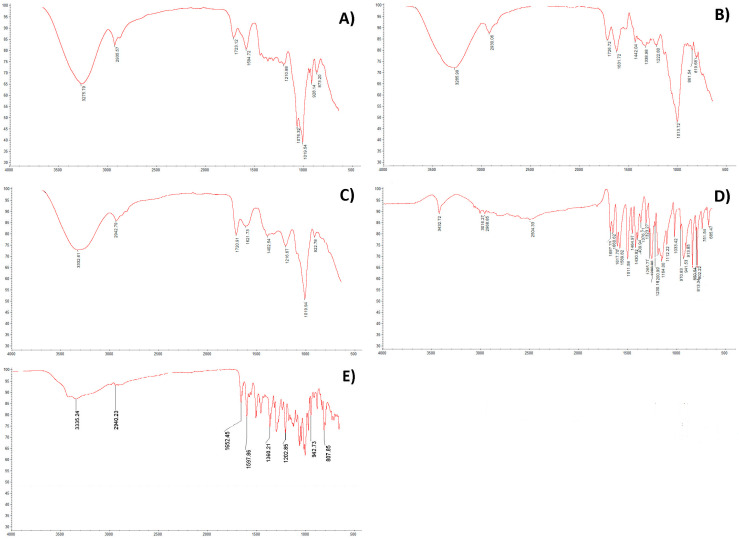
FTIR spectra in the range 4000–500 cm^−1^ of polyphenolic extracts: (**A**) persimmon, (**B**) kiwifruit, (**C**) pomegranate wines, (**D**) ferulic acid, and (**E**) rutin. For each point, 3 samples were analyzed and are shown in the figure. *y*-axis = transmittance (%), *x*-axis = wavelength (cm^−1^).

To compare the differences and similarities of polyphenol peaks, which correspond to stretching vibrations of some functional groups, the FTIR spectrum of three investigated wines, ferulic acid, and rutin between the region of 4000 and 500 cm^−1^ were combined in Figure 1. A FTIR spectral analysis of wine extracts reported similar absorption bands as obtained for the standards ferulic acid and rutin. Persimmon (3276 cm^−1^), pomegranate (3333 cm^−1^), and kiwifruit (3286 cm^−1^) wines (Figure 1A–C) showed one prominent characteristic peak, similar to the peaks observed in ferulic acid (3433 cm^−1^) and in rutin (3422 cm^−1^). Therefore, spectral bands in the range 3500–3300 cm^−1^ can be attributed to the stretching vibrations of the O–H groups, characteristic of polyphenolic extracts. In this spectral range, polyphenolic extracts had vibration bands similar to acids, as in ferulic acid (Figure 1D), however, a number of vibrational contributions of the –OH groups appeared [17]. Peaks (cm^−1^) at 2969, 2943, and 2930 appeared in ferulic acid, pomegranate, and kiwifruit wines. Rutin and persimmon wine showed similar peaks at 2936 cm^−1^. The spectral bands located between 2969 and 2930 cm^−1^ could be attributed to C-H stretching vibrations, being due to the stretching vibrations of the O–H groups [18].

Strong absorption bands in the region of 1720–900 cm^−1^ corresponded to the fingerprint region (Figure 1A–C). According to Scano [19], FTIR spectra in the fingerprint region between 1750 and 950 cm^−1^ are characteristic of polyphenolic extracts in wines. A slight difference (1600–1000 cm^−1^) may be caused by the presence of protein and the gallic acid side chain in the investigated samples [18,20]. One peak (cm^−1^) nearly at the same place (1721, 1723, and 1727) appeared only in the examined wines and was also found in other reports [19], suggesting the presence of the carbonyl C=O stretching band of protonated carboxylic acid, characteristic of the galloyl unit of hydrolyzable tannins. The bands (cm^−1^) observed a 1622, 1632, 1687, and 1650 for pomegranate and kiwifruit wines, and ferulic acid and rutin, respectively (Figure 1B–E), can be associated with the aromatic C=C stretching vibrations, present in the condensed tannins, as well as C=O stretching vibrations and the presence of unsaturated bonds in flavonoid structures. Peaks between 1465 and 1410, and 1403 and 1442 allocated the carboxylic acid (O–C–O) were observed in ferulic acid, pomegranate, and kiwifruit wines, and were not found in persimmon wine samples. The peak at 1444 cm^−1^ exhibited the C=C–C of aromatic rings and was found only in kiwifruit wine (1442 cm^−1^). The intense peak at 1447 cm^−1^ was due to the C=C–C stretching, typical of aromatic systems and found in kiwifruit wine. The peaks at 1323, 1357, and 1339 cm^−1^ for ferulic acid, rutin, and kiwifruit wine, and at 1230–1200, 1211, 1217, and 1223 cm^−1^ for ferulic acid, persimmon, pomegranate, and kiwifruit wines suggested the bending vibration of the O–H group. The peak at 1324 cm^−1^ may be attributed to the O–H bend of phenols alcohols [21] and was only found in kiwifruit wine. The peak at 1224 cm^−1^ corresponded to the C–OH of phenols and was found in kiwifruit wine (1223 cm^−1^). The spectra in kiwifruit wine showed –CH bending and –CH_2_ wagging at 1339 cm^−1^. The peak at 1065 cm^−1^ can be ascribed to the C–OH group of sugars in glycosylated phenols and was shown for persimmon wine at 1076 cm^−1^. The peak at 1016 cm^−1^ can be ascribed to the phenolic C–OH and appeared in pomegranate and kiwifruit wines at 1020 and 1014 cm^−1^, respectively.

In very recent reports [2,3] of the same types of wines, the obtained results showed that FTIR spectra (1800–900 cm^−1^) of different fruit wines can be used as main indices of the year of vintage and quality of fruit wines, of wine authentication, and of its fingerprint. The findings, based on polyphenols from fruits and fruit wines, their bioactivity, and their health properties, correspond to the main shifts in the polyphenol region of the present report.

FTIR analysis can be effectively supported by monitoring fluctuations in fluorescence intensity that arise from active compounds diffusing through an exceedingly small illumination volume, which is typically the focus of a confocal microscope. Analyzing these fluctuations through a correlation analysis provides insights into the diffusion coefficients, fluorescence brightness, and concentration of these compounds. These parameters are crucial for evaluating the size, aggregation behavior, and interactions of the active compounds in fruit wines, which are significant for understanding their impact on wine stability. Thus, to broaden and complete the data from the FTIR examination, a confocal microscopy examination of the samples was performed as the next step of the analysis.

#### 2.1.3. Confocal Microscopy Analysis

**Figure 2:**
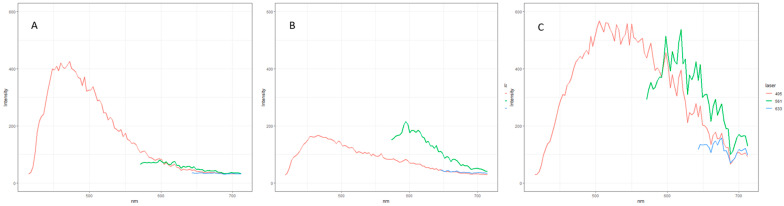
Fluorescence spectrum of fruit wines. Excitation with lasers 205, 561, and 633 nm. (**A**)—liwi wine, (**B**)—pomegranate wine, and (**C**)—persimmon wine.

The application of fluorescence correlation spectroscopy to study active compounds like carbohydrates and tannins in fruit wines could provide valuable insights into the textural properties of these wines, particularly in relation to the perception of astringency. This technique offers a non-empirical approach to modulate astringency in winemaking processes, enhancing the overall understanding of wine texture [22]. Figure 2 shows the difference in fluorescence between kiwi, persimmon, and pomegranate wines. The microscopy of wine samples showed no fluorescence from a particular structure; therefore, no images were retrieved, and the intensity graph will be further discussed. The kiwi wine sample presented high intensity with laser 405 excitation and no intensity with lasers 561 and 633 (Figure 2A). Phenolic compounds are known to be observed at a lower nm [23]. For example, intensity at a lower nm (laser 405) was high in kiwi wine (Figure 2A), followed by pomegranate (Figure 2B), while the peak in persimmon wine moved to a greater wavelength (Figure 2C); this could indicate a higher total phenolic content in kiwi and pomegranate wines than in persimmon wine. For instance, Huang et al. [24] reported values of 390 to 2300 mg/L of total phenols depending on the kiwi variety, and Akalin et al. [13] reported over 1400 mg/L of total phenols in pomegranate wine, compared with 192.78 mg/L of total phenols found in persimmon wine [25]. The intensity with laser excitation at 561 and 633 (Figure 2C) on persimmon wine samples could show anthocyanin presence due to fermentation as the alcohol percentage increases. These pigments correlate to the color present in red wine [23]. Kiwi wine is made using mature green-stage kiwifruits. Therefore, this could explain the absence of intensity with lasers 561 and 633 (Figure 2A) because of the lower content of anthocyanins. Pomegranate wine showed a lower intensity with lasers 561 and 633; this could be related to a lower anthocyanin content, which is comparable to the values reported in another study as low as 27 to 80 mg/L [26].

#### 2.1.4. Scanning Electron Microscopy Analysis

To obtain a more complete understanding of the ultrastructure of the examined wine samples, their surface morphological properties, including size, shape, and porosity, were qualitatively analyzed by scanning electron microscopy (SEM). Micrographs show morphological differences between the structure of these three types of samples that could be related to different phenolic extractability levels.

**Figure 3:**
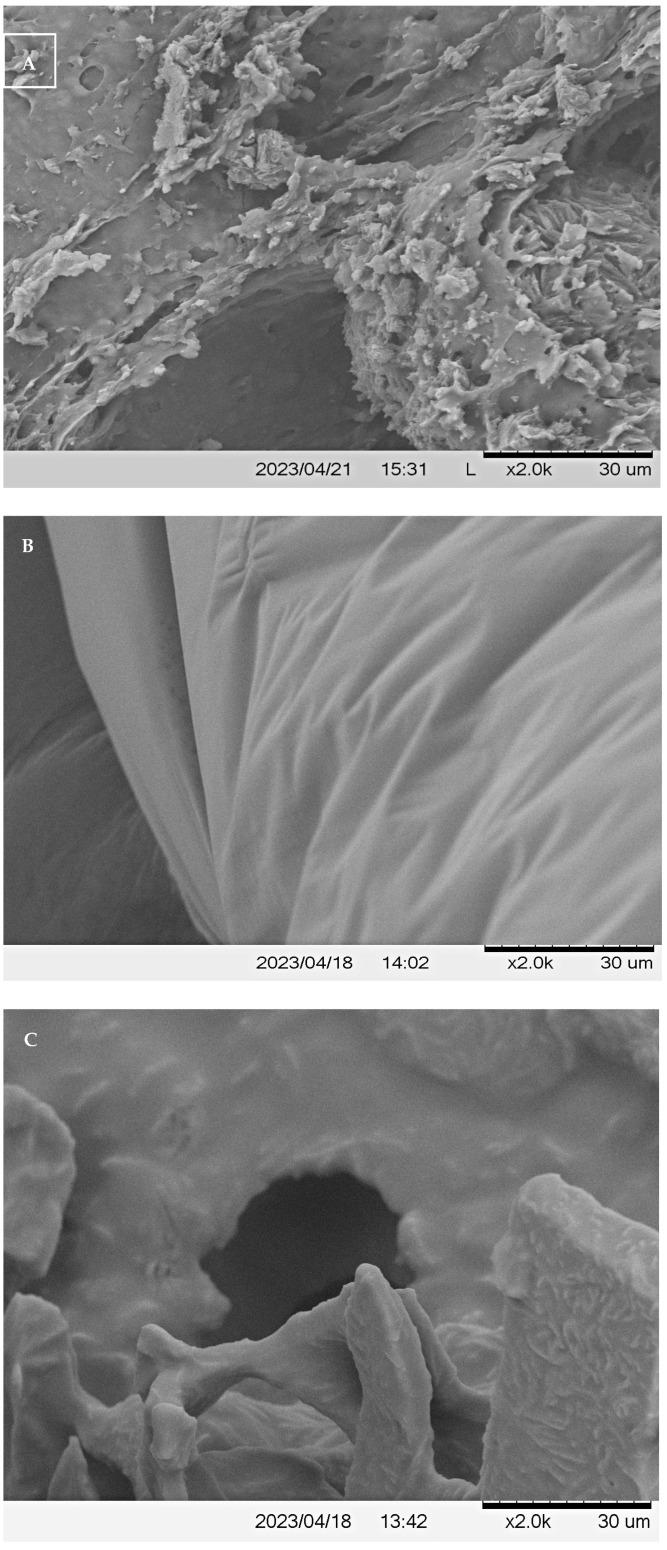
Scanning electron microscopy of freeze-dried (**A**) pomegranate wine, (**B**) kiwi wine, and (**C**) persimmon wine.

As shown in Figure 3, kiwi, persimmon, and pomegranate wines revealed different ultrastructures. Kiwi wine showed a smooth regular structure, while persimmon and pomegranate wines represented more irregular structural types, with the most irregular structure for the latter. The pomegranate wine had an irregular and lamellar structure (Figure 3A). The surface of kiwi wine displayed mostly a smooth surface with elongated indentations (Figure 3B), while the persimmon wine had lumpish particles and irregular aggregation structures (Figure 3C). The blackberry wine’s haze, rich in various polyphenolic components, displayed a layered structure, where small layers accumulate into larger ones, each with irregular areas. Some haze layers stack up to dozens of layers, and the edges of each layer appear more natural without any crystal-like formations [20]. SEM observations of fruit wines by Leng et al. [27] noted distinct polysaccharides in Cabernet Sauvignon, Cabernet Gernischt, Italian Riesling, and Moldova wine grape varieties from China, indicating unique surface morphologies for each.

#### 2.1.5. Volatile Compounds Content

**Figure 4:**
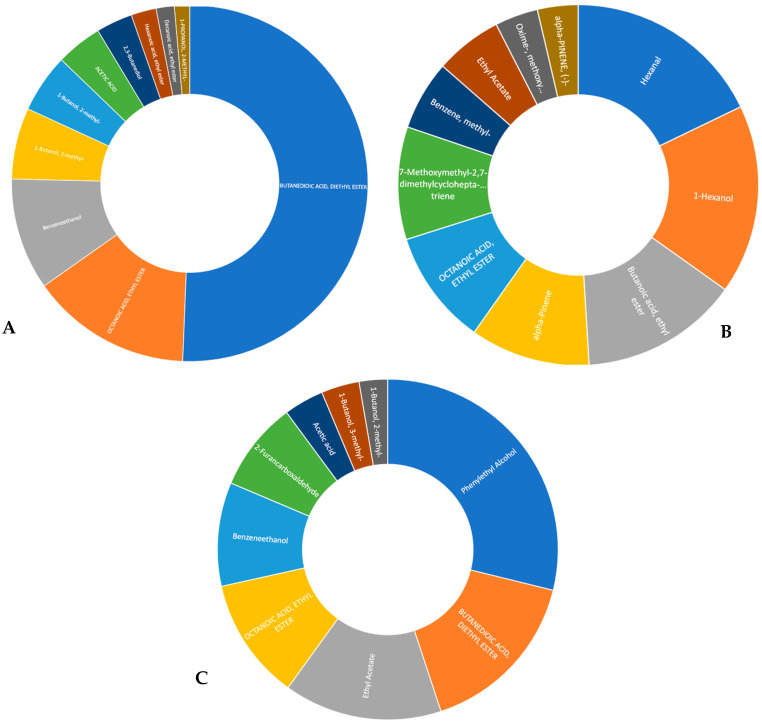
The top 10 abundant volatile compounds in fruit wines: (**A**)-pomegranate wine, (**B**)-kiwifruit wine, and (**C**)-persimmon wine.

Apart from the quantitative analysis of phenolic compounds, combined with a FTIR and microscopic analysis, this study also focused on the composition of volatile compounds (VOCs) in the examined wine samples. The adoption of a low threshold signal-to-noise ratio led to the identification of a relatively high number of chromatographic peaks, enabling the detection of trace compounds. The implementation of the GC × TOFMS technique and the peak deconvolution algorithm facilitated the tentative identification of 41 VOCs in persimmon wine, 47 VOCs in pomegranate wines, and 73 VOCs in kiwi wine, representing several chemical classes. A detailed composition of these volatile fraction compounds is gathered in the Supplementary Materials in Tables S1–S3 for pomegranate, kiwi, and persimmon wines, respectively. It was decided to present the top 10 abundant VOCs found in the volatile fraction of fruit wines in Figure 4A–C.

Butanedioic acid, diethyl ester, is a compound identified in all the examined wine samples, is primarily produced during the fermentation process when yeast metabolizes sugars. It contributes to a fruity aroma, enhancing the complexity and balance of a wine’s bouquet. This ester’s higher concentrations may particularly impart a unique fruity character to wines made from grapes grown in regions known for their volcanic soil, such as those in Yarden-type wines, contributing to distinctive characteristics known for their complexity, depth, and aging potential compared to fruits wines. The impact of the compound on wine quality is influenced by its interaction with other compounds in the wine, highlighting the need for further sensory analysis to fully understand its role. In all the evaluated fruit wines, ethyl acetate emerged as one of the most significant compounds, ranking third, fourth, and eighth in persimmon, pomegranate, and kiwi wines, respectively. This volatile compound is associated with sensory descriptors such as anise, ether, and pineapple perception. Additionally, decanoic acid, noted in ninth position in pomegranate wine, contributes to fatty and citrus impressions. Although present in persimmon and kiwi wines, it appeared in the 19th and 26th positions, respectively. Pomegranate wines also exhibited the presence of nonanal (fruit, grape, citrus, and rose) and octanoic acid (oily), as reported by Andreu-Sevilla et al. [28]. Our findings regarding the VOCs in pomegranate wines are consistent with those of Andreu-Sevilla et al. [28], who observed distinct volatile profiles characterized by a prevalence of ethyl octanoate. Additionally, varietal differences were noted in the relative abundance of alcohols, terpenes, aldehydes, esters, and organic acids, impacting sensory aspects such as odor, flavor, and color. Huang et al. [24] indicated that kiwi wine typically contains small amounts of aromatic components. Soufleros et al. [29] noted a higher impact of methanol on the aroma; however, we did not detect methanol in our samples, which is consistent with the findings of Huang et al. [24]. The aroma of persimmon wine is primarily derived from the persimmon fruit [15]. Additionally, higher alcohols formed during fermentation contribute to the desirable aroma of the wine. Liu et al. [15] noted that isoamyl alcohol and phenylethyl alcohol were the major higher alcohols. Esters, which contribute to the fruity aroma and sensory character of wines, include acetic acid 2-phenylethyl ester, decanoic acid ethyl ester, and 1-butanol-3-methyl-acetate.

#### 2.1.6. Anti-Inflammatory Properties

**Figure 5:**
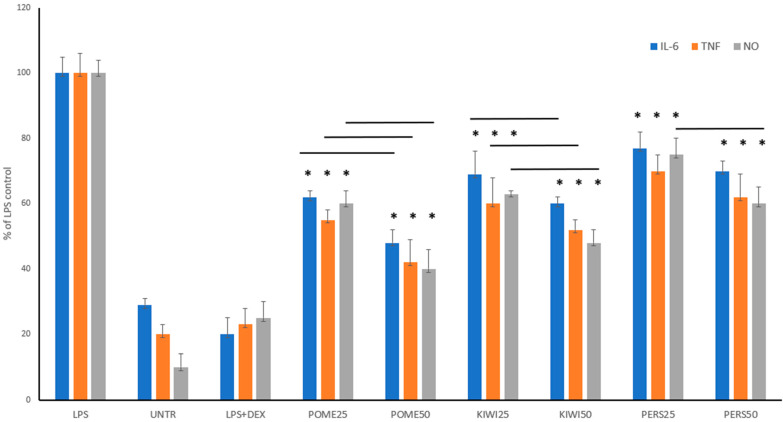
The impact of different fruits wines on the release of IL-6, TNF-α, and NO in LPS-stimulated RAW 264.7 macrophages. RAW cells were pre-treated with wines at different concentrations (the numbers in parentheses indicate concentrations in µg/mL) for 1 h, followed by the addition of 10 ng/mL of LPS to induce inflammation. Values are presented as the mean ± SD of three experiments. The results are set together with untreated RAW cells (UNTR) and cells treated with LPS and dexamethasone as the reference drug (LPS + DEX). A statistical analysis was performed using one-way ANOVA with * *p* < 0.05, against the LPS-stimulated cells. The black line indicates significant differences between the used doses. Abbreviations are as follows: POME—pomegranate wine; KIWI—kiwifruit wine. PERS—persimmon wine.

The analysis of VOCs in the examined wine samples indicated the presence of various compounds, known for interesting biological effects, including anti-inflammatory properties, such as fatty acids and their esters; terpenes, such as alpha-pinene and limonene; alcohols including 1-butanol, hexanal, 1-hexanol, octanoic acid, ethyl ester, and methyllaurate. Thus, as the ultimate step of this study, the anti-inflammatory potential of pomegranate, kiwi, and persimmon wines was determined, using an LPS-stimulated RAW 264.7 macrophage model. Three different parameters were evaluated to assess the significance of anti-inflammatory activity, namely nitric oxide, IL-6, and TNF-α release (Figure 5). All wine samples demonstrated notable anti-inflammatory effects in vitro, with pomegranate, kiwi, and persimmon wines showing decreasing efficacy in that order. Notably, dose-dependent differences were mostly indiscernible for persimmon wines. These findings correlate well with the composition of active polyphenolic compounds within these wines.

Notably, this is the first study presenting the anti-inflammatory potential of pomegranate, kiwi, and persimmon wines. Previously, only Verotta et al. [30] confirmed the anti-inflammatory properties of fermented pomegranate waste extract, rich in ellagic acid, demonstrating its safety across a wide concentration range of 5–100 µg/mL—comparable results were replicated in our study. RAW 264.7 cells were pre-incubated with this pomegranate extract at a concentration of 50 µg/mL before LPS treatment. Results showed that pre-treatment with the extracts significantly reduced the LPS-induced expression of TNF-α, IL-1β, and iNOS. Furthermore, the anti-inflammatory activities of kiwi fruits and their by-products are linked to their polyphenolic compounds, in addition to other antioxidants present in the fruit of *Actinidia arguta* [31]. For persimmon vinegar, Hosseininejad et al. [32] have also confirmed its anti-inflammatory properties.

## 3. Materials and Methods

### 3.1. Materials

Kiwifruits and persimmon were collected from South Korea, and pomegranates from Israel, in 2022. Fruit wines were crafted using methods previously detailed by our group [33], employing the *S. bayanus* Lalvin strain EC1118 for fermentation. This strain can be used in a wide range of applications such as sparkling wines, fruit wines, and ciders. This yeast was grown on a YPD agar medium and maintained at 4 °C until use. Kiwi, pomegranate, and persimmon fruit pulps were prepared by adding tartaric acid (4.5 g), tannin (0.45 g), and ammonium phosphate (6 g) before fermentation. The fruit was treated with pectinase for 24 h at room temperature and adjusted for sugar and acid levels.

Fermentation involved activating 3 g of yeast in 15 mL of warm water (40 °C), followed by fermentation at 14 °C for two weeks. The wine was then transferred to a secondary jar to enhance quality and prevent sluggish fermentation, aged for another four weeks, and finally racked into a new jar to age for three months, ensuring residual sugars were below 5%. The wine was then filtered, bottled, and stored at 14 °C until analysis. All investigated wines were prepared in South Korea according to the above procedures under industrial conditions, which were controlled. The wine samples were evaporated until the last drop of ethanol, then frozen with liquid nitrogen and lyophilized for 48 h using a lyophilizer (Virtis model 10–324, Midland, ON, Canada).

Fresh or dry samples obtained by lyophilization were stored until the analysis in a refrigerator at −20 °C.

### 3.2. HPLC Analysis

Lyophilized wine samples (50 mg each) were dissolved in 1 mL of dimethyl sulfoxide (DMSO). Polyphenols in the wine samples were determined using an HPLC method, as previously outlined in reference [34]. The HPLC system comprised a Dionex 100 (DionexSoftron GmbH, Germering, Germany) with a Photodiode 100 detector. An injection volume of 20 μL of each extract was applied to a Hypersil Gold (C-18) column (5 μm, 250 × 4.6 mm, Thermo Scientific, Runcorn, UK) and analyzed with a mobile phase consisting of 1% formic acid (A) and acetonitrile (B) in a gradient mode of 5–60% B over 60 min. For peak identification, the retention times and UV spectra (λ = 254, 285 nm) of obtained peaks were compared with those of the standards. The quantification of phenolic acids (chlorogenic, caffeic, p-coumaric, gallic, and protocatechuic acid) was conducted using standard curves derived from reference substance solutions. All analyses were conducted in triplicate, and the mean values were expressed as mg/g of wine lyophilizates.

### 3.3. FTIR Data Collection

The polyphenol dry extracts of wines and the standards were used for FTIR measurements. A Nicolet iS 10 Fourier transform infrared (FTIR) Spectrometer (ThermoScientific Instruments LLC, Madison, WI, USA), with a smart iTRTM attenuated total reflectance (ATR) accessory was used to record IR spectra. Air was used as a reference background; a new background spectrum was recorded every ten analyses. The sample was placed directly on the platform, uniform pressure was applied, and the spectra were recorded as the average of 24 scans at 4 cm^−1^ resolution. Between samples, the platform was cleaned with isopropyl alcohol. Three replicates were used for each sample. On average, the collection of the three replicates for each sample took 3–5 min. Ferulic acid and rutin were used as references [2,3,17].

### 3.4. Confocal Microscopy Methodology

Aliquots of freeze-dried particles from kiwi, pomegranate, and persimmon wines were dispersed on the surface of microscope slides and examined with a SP5 confocal microscope system (Leica Microsystems—Wetzlar, Germany) using a 100× objective oil-immersion lens and 405, 561, and 633 nm laser lines. The samples’ fluorescence emission was measured and compared from 425 to 750, 573 to 775, and 645 to 766 nm as emission spectra for each laser, respectively. A 3-channel Z-stack image was acquired for each, and a maximum projection image of particle fluorescence was created for analysis.

### 3.5. Surface Morphology Analysis

With the purpose of studying the structural features of fruit wines, a scanning electron microscopy (SEM; S-3400N II, Hitachi, High-Tech America Inc., Schaumburg, IL, USA) analysis was conducted. Aliquots of freeze-dried powder were mounted to aluminum stubs using conductive carbon tape. Samples were sputter-coated with gold using a Desk IV Sputter Coater (Denton Vacuum, Moorestown, NJ, USA) and viewed at 5 kV in the secondary electron imaging mode. Digital images of the particles were collected and visually compared.

### 3.6. GC-TOFMS Analysis of Volatile Compounds

Each wine sample (4.0 ± 0.1 g) was placed in a 20 mL vial which was then sealed with a PTFE-lined silicone septum. The extraction of volatile compounds from the wines was achieved through headspace solid-phase microextraction (HS-SPME). Prior to extraction, the samples were heated at 40 °C for 3 min and agitated at 700 rpm. Before each injection, the fiber was desorbed at 250 °C for 3 min in a desorption unit. Extraction was performed at 40 °C for 47 min using a divinylbenzene/carboxen/polydimethylsiloxane (DVB/CAR/PDMS) SPME fiber of 50/30 μm thickness and 1 cm of length (Merck Co., New York, NY, USA). After extraction, the fiber was withdrawn from the vial and transferred to the injector of a gas chromatograph for thermal desorption of the analytes at 250 °C for 280 s and then subjected to a second desorption in the unit at 250 °C for 5 min.

The GC system comprised an Agilent 6890A gas chromatograph (Agilent Technologies, Palo Alto, CA, USA) coupled with a Pegasus IV time-of-flight mass spectrometer (LECO Corp., St. Joseph, MI, USA). The column set consisted of a 30 m × 0.25 mm × 1 μm column with an Equity1 stationary phase (Supelco Co., Bellefonte, PA, USA) and a 0.5 m × 0.1 mm guard column (Restek, Bellefonte, PA, USA). The separation of sample components was achieved using an optimized temperature program: the initial temperature was set at 28 °C and maintained for 2 min, then ramped at 4 °C/min to 190 °C, at 8 °C/min to 210 °C, and at 12 °C/min to 250 °C, where it was held for 2 min. The total analysis time was approximately 47.8 min. The injector was operated at 250 °C in splitless mode. Helium (N6.0 grade) was utilized as the carrier gas at a constant flow rate of 1.0 mL/min. The transfer line was maintained at 250 °C. The ion source temperature was 250 °C, and the detector voltage was set to −1647 V. Ions in the *m*/*z* 35–450 range were analyzed at a relatively slow data acquisition rate of 10 spectra/s to increase method sensitivity. To ensure consistent sample preparation and dosing conditions, the apparatus was equipped with a multitask autosampler MPS II (Gerstel Co., Mülheim an der Ruhr, Germany).

Manual data processing was conducted using the peak deconvolution program implemented in the ChromaTOF software (LECO Corp., version 4.51.6.0). Tentative identification of analytes was achieved through an MS library search using the NIST 2011 and Wiley 2010 libraries. A key criterion for peak selection was a comparative parameter that described the similarity of a particular chromatographic peak’s mass spectrum with the spectrum from the database. The examples of GC-TOFMS chromatograms of the volatile fraction wine profiles in TIC mode are presented in the Supplementary Material as Figures S1–S3.

### 3.7. Determination of NO, IL-6, and TNF-α Release

Before conducting anti-inflammatory tests, the toxicity of the investigated samples was assessed using RAW 264.7 macrophages. The cells were seeded in 96-well plates at a density of 1.5 × 10^5^ cells per well and exposed to varying concentrations of fruit wines (0–100 µg/mL) for 24 h. The viability of the cells was then evaluated using the MTT assay, with all tests conducted in triplicate. Results were presented as the percentage of cell viability, expressed as the mean ± standard deviation. Based on these results, concentrations of 25 and 50 µg/mL of the freeze-dried wines were selected as non-toxic and used in subsequent anti-inflammatory tests.

In the anti-inflammatory experiments, RAW 264.7 cells were seeded in 96-well plates at the same density and pre-treated with the wine samples of the selected concentrations or dexamethasone (reference drug, 0.5 µg/mL) for one hour. This was followed by exposure to 10 ng/mL of LPS to initiate inflammation, consistent with previous methods [35]. The cells were incubated for 24 h and the supernatants were then analyzed to measure nitric oxide production using a Griess Reagent Kit (Promega Corporation, Madison, Winooski, VT, USA), and levels of cytokines such as TNF-α and IL-6 were analyzed using Human ELISA kits (Bioassay Technology Laboratory, Shanghai, China) as per the supplier’s guidelines. These assays were also performed in triplicate, and absorbance was read on a microplate reader (SynergyTM HT—BioTek Instruments Inc., Winooski, VT, USA). The outcome of these assays was expressed as a percentage of the control values.

### 3.8. Statistical Analysis

The data were analyzed by a one-way ANOVA followed by a post hoc test using the STATISTICA v.13.3. package (TIBCO Software Inc., Palo Alto, CA, USA).

## 4. Conclusions

The applied combination of quantitative HPLC and FTIR analyses, supported by confocal microscopy examination, can provide a thorough examination of exotic fruit wines’ polyphenolic fraction. The examined kiwi, persimmon, and pomegranate wines were characterized by varied polyphenolic content, and also differ in their textural properties. The wines were also rich in volatile compounds, which may translate into their interesting anti-inflammatory potential. In terms of polyphenols and anti-inflammatory potential, pomegranate and kiwifruit wines exhibited greater benefits. However, kiwifruit wines were the least rich in the profile of volatile compounds. Further analyses are necessary, particularly concerning the use of other microorganisms in the fermentation process and non-*Saccharomyces* strain methods. Research in this direction should be continued, as exotic fruit wines are a valuable, antioxidant-rich element of the human diet.

## Figures and Tables

**Table 1:** Concentration of phenolic acids in freeze-dried fruit wines [mg/g], n = 3.

Phenolic Acids	Pomegranate Wine	Kiwi Wine	Persimmon Wine
chlorogenic acid	3.16 ± 0.30 ^ab^	0.49 ± 0.10 ^ac^	0.10 ± 0.04 ^bc^
gallic acid	7.99 ± 0.23 ^ab^	1.78 ± 0.10 ^ac^	0.44 ± 0.11 ^bc^
caffeic acid	1.09 ± 0.25 ^ab^	0.10 ± 0.01 ^ac^	0.06 ± 0.00 ^bc^
*p*-coumaric acid	0.09 ± 0.02	<LOD	<LOD
protocatechuic acid	0.22 ± 0.03 ^ab^	0.76 ± 0.10 ^ac^	0.05 ± 0.02 ^bc^
sum of phenolic acids	12.5	3.13	0.65

The same letters in superscript indicate significant differences in the results within the row; levels of significance *p* < 0.05.

## Data Availability

The original contributions presented in the study are included in the article/Supplementary Material, further inquiries can be directed to the corresponding author.

## Supplementary Materials

The supporting information can be downloaded at: https://www.mdpi.com/article/10.3390/ijms25115627/s1.
