# Supplementary material for: Physicochemical and Volatile Compounds Analysis of Fruit Wines Fermented with Saccharomyces cerevisiae: FTIR and Microscopy Study with Focus on Anti-Inflammatory Potential
Source: Int J Mol Sci. 2024 May 22;25(11):5627. doi: 10.3390/ijms25115627 (PMC11172100; doi:10.3390/ijms25115627)
Supplement: Supplementary file 1 [file ijms-25-05627-s001.zip › ijms-3000283-supplementary.pdf]

Table S1. Compounds identified in the volatile fraction of pomegranate wines using HS-SPME/GC -TOFMS.

Index	Name	CAS	Area	%Area	Similarity	RT	Unique m
1	BUTANEDIOIC ACID, DIETHYL ESTER	123-25-1	952,230,721	39.849	978	1413.8	101
2	OCTANOIC ACID, ETHYL ESTER	106-32-1	274,031,010	11.468	929	1476.3	88
3	Benzeneethanol (CAS)	60-12-8	190,471,433	7.9708	958	1274.7	122
4	Ethyl Acetate	141-78-6	122,788,803	5.1384	964	302.8	70
5	1-Butanol, 2-methyl- (CAS)	137-32-6	101,308,325	4.2395	959	470.3	56
6	ACETIC ACID	64-19-7	77,877,852	3.2590	945	335.3	45
7	2,3-Butanediol	513-85-9	61,842,275	2.5879	952	551.0	45
8	Hexanoic acid, ethyl ester (CAS)	123-66-0	42,536,708	1.7801	956	1031.1	88
9	Decanoic acid, ethyl ester (CAS)	110-38-3	32,155,236	1.3456	924	1872.2	88
10	1-PROPANOL, 2-METHYL-	78-83-1	26,452,239	1.1070	951	320.4	41
11	1-Butanol, 3-methyl-	123-51-3	13,663,789	0.5718	969	464.5	46
12	2,3-BUTANEDIOL	513-85-9	12,570,275	0.52604	956	574.2	45
13	Furan, 2,2'-[oxybis(methylene)]bis- (CAS)	4437-22-3	10,997,706	0.46023	812	796.0	81
14	Pentadecylamine	2570-26-5	9,568,365	0.40041	806	1414.6	44
15	1-Butanol, 2-methyl-, acetate (CAS)	624-41-9	7,984,600	0.33414	937	755.0	43
16	ACETIC ACID, 2-PHENYLETHYL ESTER	103-45-7	6,825,290	0.28562	923	1571.6	104
17	1-BUTANOL, 3-METHYL-, ACETATE	123-92-2	6,538,460	0.27362	944	748.5	55
18	Butyrolactone	96-48-0	6,331,008	0.26494	953	759.7	41
19	2-Furancarboxaldehyde (CAS)	98-01-1	6,277,782	0.26271	947	618.4	95
20	Benzene, methyl- (CAS)	108-88-3	6,153,417	0.25751	943	519.5	92
21	Nonanoic acid, ethyl ester (CAS)	123-29-5	4,444,182	0.18598	914	1678.3	88
22	1-Hexanol (CAS)	111-27-3	4,283,711	0.17926	915	737.4	55
23	Butanoic acid, ethyl ester (CAS)	105-54-4	2,679,249	0.11212	902	584.1	71
24	Benzaldehyde (CAS)	100-52-7	2,566,402	0.10740	955	909.3	77
25	1,3-DIOXOLANE, 2,4,5-TRIMETHYL-	3299-32-9	2,379,630	0.099582	941	453.3	101
26	2-Butanone, 3,3-dimethyl-1-(methylsulfonyl)-, (R)	39184-59-3	2,348,135	0.098264	812	1272.1	45
27	Propanoic acid, 2-methyl-, ethyl ester (CAS)	97-62-1	2,091,186	0.087511	909	507.2	71
28	1H-Purin-6-amine, [(2-fluorophenyl)methyl]- (CAS)	74421-44-6	1,968,548	0.082379	811	2624.7	73
29	Propanoic acid, 2-methyl-, 1-(1,1-dimethylethyl)-, (R)-	74381-40-1	1,954,594	0.081795	851	2234.1	71

30	Oxirane, phenyl- (CAS)	96-09-3	1,883,098	0.0788 03	923	1095.1	91
31	Pentanoic acid, 2-hydroxy-4-methyl-, ethyl ester	10348-47-7	1,620,598	0.0678 18	865	1163.9	69
32	Octanoic acid	124-07-2	1,479,233	0.0619 02	895	1430.1	60
33	Ethyl hydrogen succinate	1070-34-4	1,438,729	0.0602 07	881	1395.3	101
34	Tridecane (CAS)	629-50-5	1,367,783	0.0572 38	923	1515.4	57
35	BUTANOIC ACID, 3-METHYL-, ETHYL ESTER	108-64-5	1,259,745	0.0527 17	887	699.7	88
36	Hexanoic acid (CAS)	142-62-1	1,144,310	0.0478 87	912	1001.0	60
37	1-Hexanol, 2-ethyl- (CAS)	104-76-7	1,137,573	0.0476 05	908	1108.8	57
38	Benzene, 1,2-dichloro- (CAS)	95-50-1	924,765	0.0386 99	874	1037.4	146
39	Benzeneacetic acid, ethyl ester	101-97-3	851,976	0.0356 53	933	1545.9	65
40	Nonanal (CAS)	124-19-6	516,595	0.0216 18	896	1263.1	56
41	Isobutyl acetate	110-19-0	296,366	0.0124 02	856	533.0	56
42	ethyl 9-decenoate	67233-91-4	192,549	0.0080 58	812	1850.2	88
43	Octanoic acid, methyl ester (CAS)	111-11-5	178,676	0.0074 77	835	1315.4	87
44	vitispirane	65416-59-3	115,827	0.0048 47	842	1663.4	121
45	Hexadecanoic acid, ethyl ester (CAS)	628-97-7	88,682	0.0037 11	851	2227.2	101
46	o-Xylene	95-47-6	55,078	0.0023 05	896	787.6	106
47	2-Furancarboxylic acid, ethyl ester	614-99-3	40,452	0.0016 93	839	1125.3	96

Table S2. Compounds identified in the volatile fraction of kiwi wine using HS-SPME/GC -TOFMS.

Index	Name	CAS	Area	%Area	Similarity	RT	unique m
1	Hexanal	66-25-1	49692160.0	8.2725	945	546.1	41
2	1-Hexanol	111-27-3	47261066.0	7.8678	942	685.1	42
3	Butanoic acid, ethyl ester	105-54-4	39706810.0	6.6102	952	558.7	88
4	à-Pinene	80-56-8	29682492.0	4.9414	935	820.9	93
5	OCTANOIC ACID, ETHYL ESTER	106-32-1	137,015,505.0	4.834	929	1476.3	88
6	7-Methoxymethyl-2,7-dimethylcyclohepta-1,3,5-triene	73992-48-0	28117867.0	4.6809	830	1579.9	119
7	Benzene, methyl-	108-88-3	17468754.0	2.9081	952	502.3	91

Index	Name	CAS	Area	%Area	Similarity	RT	unique m
8	Ethyl Acetate	141-78-6	61,394,401.5	2.769	964	302.8	70
9	Oxime-, methoxy-phenyl-	0-00-0	10984517.0	1.8286	803	771.4	151
10	à-PINENE, (-)-	80-56-8	12819317.0	1.7157	931	819.8	93
11	Benzene, (1,1-dimethyldecyl)-	27854-40-6	11418053.0	1.5282	799	1579.8	119
12	Octane	111-65-9	8805001.0	1.4658	876	581.1	43
13	l-Limonene	5989-54-8	8302419.0	1.3821	913	977.8	68
14	CYCLOHEXENE, 1-METHYL-4-(1-METHYLETHENYL)-	138-86-3	6735627.0	1.2167	907	977.3	68
15	2,3-Butanediol	513-85-9	30,921,137.5	1.194	952	551.0	45
16	2-PHENYL-3-(P-TOLYL)-BUTANE	0-00-0	6288111.0	1.1358	831	1579.7	119
17	o-Cymene	527-84-4	6738140.0	1.1217	940	962.1	134
18	1-Pentanol	71-41-0	5503864.0	0.91625	917	512.2	42
19	Hexanoic acid, ethyl ester	123-66-0	21,268,354.0	0.890	956	1031.1	88
20	Cyclohexene, 3-methyl-6-(1-methylethyl)-	5256-65-5	6282824.0	0.84088	899	970.0	95
21	Benzene, ethyl-	100-41-4	4434752.0	0.80107	925	669.2	91
22	p-Xylene	106-42-3	4700322.0	0.78248	813	686.3	91
23	1-Pentanol	71-41-0	5718434.0	0.76534	926	508.1	42
24	METHYL 2-METHOXYPROPANOATE	17639-76-8	4208667.0	0.76023	797	667.3	59
25	Cyclohexane, 1-(1,5-dimethylhexyl)-4-methyl-	29799-19-7	4316656.0	0.71861	841	1578.4	97
26	Decanoic acid, ethyl ester	110-38-3	16,077,618.0	0.673	924	1872.2	88
27	á-Pinene	127-91-3	4004506.0	0.66665	929	891.9	93
28	METHYLLAURATE	0-00-0	4826801.0	0.64601	846	668.5	91
29	1,1'-Bicycloheptyl	23183-11-1	3868816.0	0.64406	856	922.5	55
30	Cyclohexane, 1-methyl-4-(1-methylethylidene)-	1124-27-2	3722653.0	0.61973	839	907.5	95
31	o-Xylene	95-47-6	3363624.0	0.55996	939	727.3	91
32	Cyclohexane, 1-methyl-4-(1-methylethyl)-, trans-	1678-82-6	4136298.0	0.55359	853	896.5	97
33	Ethene, tetrachloro-	127-18-4	3143173.0	0.52326	914	581.6	166

Index	Name	CAS	Area	%Area	Similarity	RT	unique m
34	PENTADECANE	629-62-9	2790602.0	0.46456	917	1653.3	57
35	2-Hexenal, (E)-	6728-26-3	3010704.0	0.40295	918	637.1	83
36	1-Butanol, 3-methyl-	123-51-3	6,831,894.5	0.386	969	464.5	46
37	Bicyclo[3.1.1]heptane, 6,6-dimethyl-2-methylene-, (1S)-	18172-67-3	1984496.0	0.35847	874	891.2	93
38	Tetradecane	629-59-4	2125293.0	0.35381	880	1528.0	57
39	Octane	111-65-9	2520829.0	0.33738	787	578.2	85
40	Methyl (2R)-2-Hydroxybutanoate	0-00-0	2443820.0	0.32708	789	667.7	59
41	Butanoic acid, hexyl ester	2639-63-6	1947640.0	0.32423	874	1217.4	71
42	Nonane	111-84-2	1923629.0	0.32023	919	763.1	57
43	3-Hexen-1-ol	544-12-7	1672291.0	0.30207	866	658.3	41
44	Pentadecane	629-62-9	1613323.0	0.29142	931	1653.1	57
45	2-Hexenal	505-57-7	1556061.0	0.28108	907	640.0	69
46	CYCLOPENTANE, 1-METHYL-3-(2-METHYLPROPYL)-	29053-04-1	1686278.0	0.28072	816	1592.0	97
47	Heptadecane, 2-methyl-	1560-89-0	2040440.0	0.27309	900	1653.3	57
48	2,3-BUTANEDIOL	513-85-9	6,285,137.5	0.263	956	574.2	45
49	Cyclohexane, 1-isopropyl-3-methyl-, (E)-	0-00-0	1572937.0	0.26185	889	897.6	81
50	1-Methyl-4-(1-methylethyl)-cyclohexane	99-82-1	1296430.0	0.23418	873	921.4	41
51	pentadecane	629-62-9	1718558.0	0.23001	891	1527.9	57
52	Propanoic acid, 2-methyl-, ethyl ester	97-62-1	1268824.0	0.22919	926	490.8	71
53	TRANS-á-IONON-5,6-EPOXIDE	23267-57-4	1258549.0	0.22734	908	479.7	40
54	Heptadecane	629-78-7	1278154.0	0.21278	824	1501.4	71
55	Decane	124-18-5	1152525.0	0.20819	890	937.7	57
56	Butyrolactone	96-48-0	1145793.0	0.19075	911	736.2	42
57	3-Pentanone, 2-methyl-	565-69-5	1054952.0	0.19056	922	470.5	57

Index	Name	CAS	Area	%Area	Similarity	RT	unique m
58	Tetrachloroethylene	127-18-4	1045589.0	0.18887	817	579.6	164
59	Cyclohexanecarbonitrile	766-05-2	1363113.0	0.18244	755	897.3	54
60	Butanoic acid, hexyl ester	2639-63-6	1357092.0	0.18163	892	1217.3	89
61	Tetradecane	629-59-4	1077070.0	0.1793	923	1395.0	85
62	2(3H)-Furanone, dihydro-	96-48-0	982631.0	0.1775	935	723.9	42
63	OCTANE, 2,6-DIMETHYL-	2051-30-1	1286003.0	0.17212	861	825.0	57
64	1-Propanamine	107-10-8	1033444.0	0.17204	885	541.2	59
65	1-BUTANOL, 3-METHYL-	123-51-3	1,034,113.0	0.172%	939	460.5	55
66	Camphene	79-92-5	983752.0	0.16377	855	844.8	93
67	Furan, 2,5-diethyltetrahydro-	41239-48-9	1193885.0	0.15979	825	728.8	81
68	Cyclohexane, 1,1'-(1-methylethylidene)bis-	54934-90-6	1131304.0	0.15141	864	1753.0	69
69	Cyclohexene, 1-methyl-4-(1-methylethyl)-, (R)-	1195-31-9	833142.0	0.1387	848	970.7	138
70	Oxalic acid, butyl cyclohexylmethyl ester	0-00-0	744092.0	0.099588	818	1592.0	97
71	1-Butanol, 3-methyl-	123-51-3	608,914.5	0.081	887	456.2	55
72	Hexanoic acid, ethyl ester	123-66-0	389,504.5	0.052%	870	910.1	88
73	Octanoic acid	124-07-2	739,616.5	0.041	895	1430.1	60

Table S3. Compounds identified in the volatile fraction of persimmon wine using HS-SPME/GC -TOFMS.

Index	Name	CAS	Area	%Area	Similarity	RT	unique m
1	Phenylethyl Alcohol	60-12-8	461090882	31.935	958.0	1273.0	91
2	BUTANEDIOIC ACID, DIETHYL ESTER	123-25-1	1215465341	17.804	955.5	1416.1	147
3	Ethyl Acetate	141-78-6	1157851207	16.655	960.0	303.6	71
4	OCTANOIC ACID, ETHYL ESTER	106-32-1	183910458	12.738	929.0	1476.0	88
5	Benzeneethanol	60-12-8	381139926	10.884	963.0	1271.5	91
6	2-Furancarboxaldehyde	98-01-1	311482241	9.4461	954.0	617.7	96

Index	Name	CAS	Area	%Area	Similarity	RT	unique m
7	Acetic acid	64-19-7	159905954	4.2117	953.5	350.3	45
8	1-Butanol, 3-methyl-	123-51-3	263994708	4.0338	957.0	464.4	69
9	1-Butanol, 2-methyl-	137-32-6	121681810	3.06805	884.5	470.6	56
10	Benzeneacetic acid, ethyl ester	101-97-3	30409409	2.1062	955.0	1546.1	91
11	1-PROPANOL, 2-METHYL-	78-83-1	58878394	1.618345	929.5	320.7	41
12	Hexanoic acid, ethyl ester	123-66-0	46921958	1.5287	940.0	1031.5	88
13	Oxime-, methoxy-phenyl-	0-00-0	15143685	1.0489	833.0	832.9	133
14	Propanoic acid, 2-hydroxy-, ethyl ester, (S)-	687-47-8	35874230	1.0245	947.0	608.5	45
15	Ethyl sorbate	2396-84-1	11791470	0.81668	918.0	1241.7	67
16	Butyrolactone	96-48-0	27381038	0.67308	959	764.5	42
17	CIS-4-HYDROXY-3-METHYLOCTANOIC ACID LACTONE	0-00-0	27149364.0	0.58471867	905	1647.1	99
18	Benzeneacetic acid, ethyl ester	101-97-3	18283392	0.52213	945	1545.7	91
19	DECANOIC ACID, ETHYL ESTER	110-38-3	14504785	0.479819	907	1871.8	88
20	2,3-BUTANEDIOL	513-85-9	11848090	0.410298	906	578.4	45
21	Benzeneacetaldehyde	122-78-1	5851919	0.4053	920	1094.8	91
22	N-benzylidene-dimethylammonium chloride	0-00-0	5653733	0.39158	920	909.5	105
23	2-Furancarboxylic acid, ethyl ester	614-99-3	18573414	0.390725	942	1125.1	112
24	BUTANOIC ACID, ETHYL ESTER	105-54-4	5566455	0.38553	941	584.6	71

Index	Name	CAS	Area	%Area	Similarity	RT	unique m
25	TRANS-4-HYDROXY-3-METHYLOCTANOIC ACID LACTONE	0-00-0	13109704	0.37438	937	1686.8	99
26	Propanoic acid, ethyl ester	105-37-3	4659134	0.32269	923	423.2	57
27	Propanoic acid, 2-methyl-, ethyl ester	97-62-1	10127431	0.2870845	903	507.4	43
28	2-NONANOL	628-99-9	3782144	0.26195	705	1271.4	45
29	ACETIC ACID, 2-PHENYLETHYL ESTER	103-45-7	8452083	0.2290005	908	1571.7	104
30	Benzeneacetaldehyde	122-78-1	5634853	0.16092	901	1094.4	91
31	2,3-Butanediol	513-85-9	5485296	0.15665	915	563.0	45
32	Succinic acid, butyl ethyl ester	0-00-0	4697813	0.128162	874	1719.8	101
33	1,1-Diethoxypropanal	0-00-0	4954956	0.1231855	886	844.5	47
34	1-Butanol, 3-methyl-, acetate	123-92-2	4177303	0.119499	935	749.0	61
35	diethyl hydroxybutanedioate	0-00-0	4551858	0.1145575	857	1586.0	71
36	Octanoic acid, ethyl ester	106-32-1	3906018	0.11155	888	1474.1	88
37	Butanoic acid, 2-methyl-, ethyl ester	7452-79-1	3748837	0.1087175	916	694.3	102
38	Benzaldehyde	100-52-7	3574503	0.10208	955	909.1	105
39	Pentanoic acid, 2-hydroxy-4-methyl-, ethyl ester	10348-47-7	3915654	0.097792	866	1164.1	69
40	ethyl 3-methylbutyl butanedioate	0-00-0	6416940	0.08113655	864	1911.7	129
41	2,4-Hexadienoic acid, ethyl ester	110318-09-7	3695136	0.061217	878	1234.1	67

Figures S1-S3 show the GC-TOFMS chromatograms of the volatile fraction wine profiles in TIC mode.

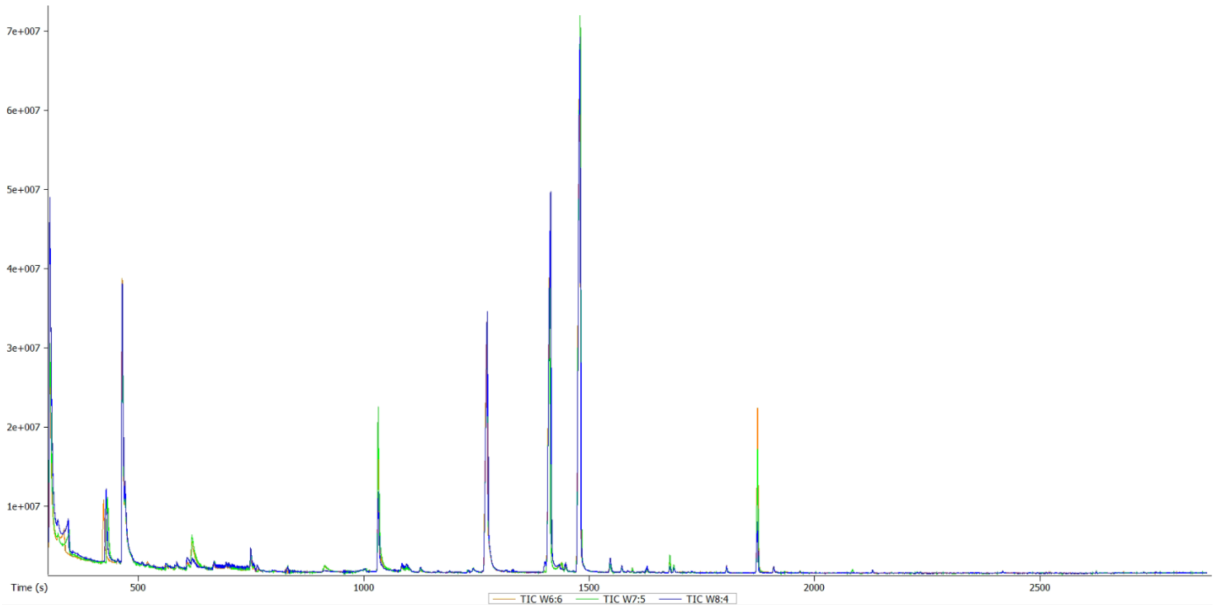

Figure S1. GC-TOFMS chromatograms of the volatile fraction profiles of pomegranate wines in TIC mode.

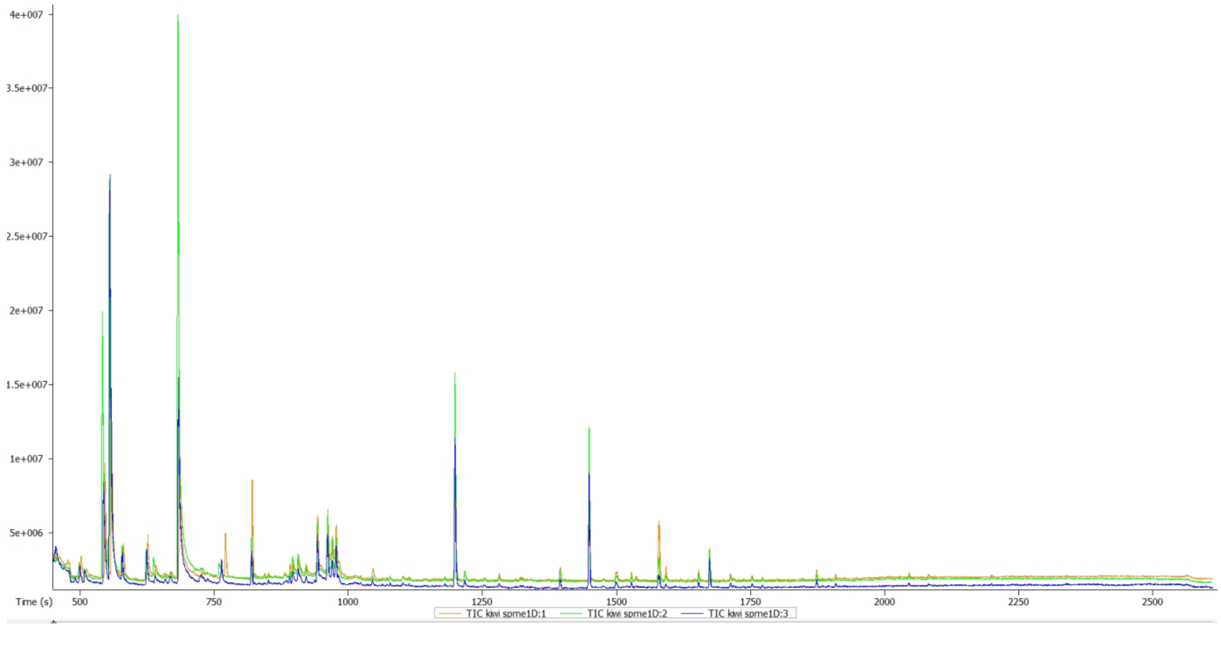

Figure S2. GC-TOFMS chromatograms of the volatile fraction profiles of kiwi wine in TIC mode.

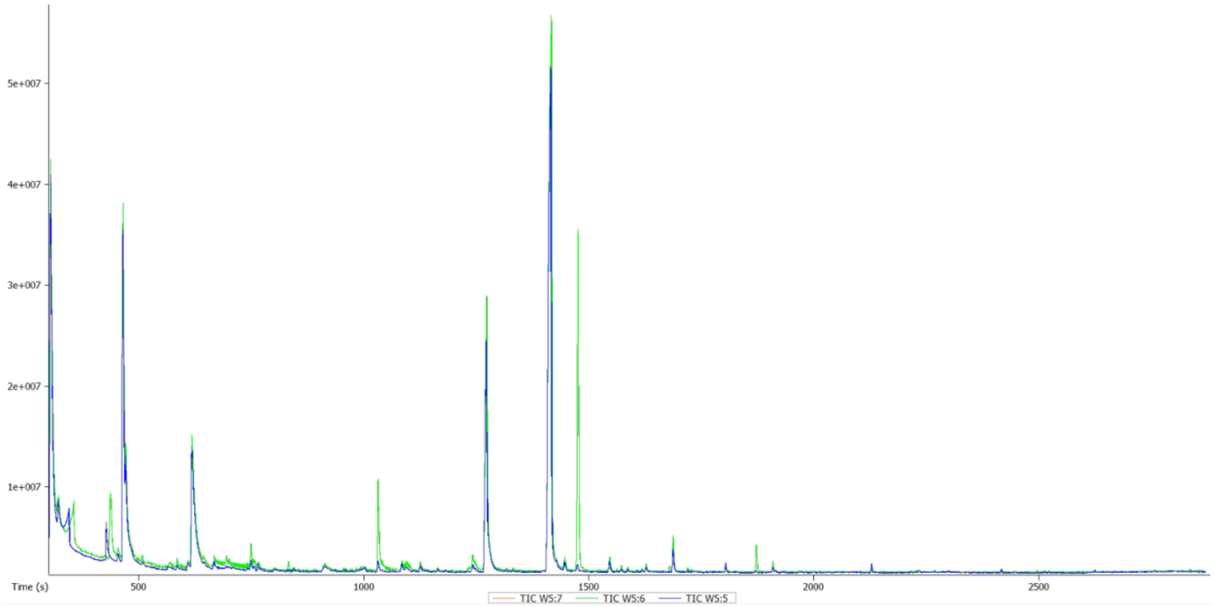

Figure S3. GC-TOFMS chromatograms of the volatile fraction profiles of persimmon wine in TIC mode.
